# Splenic artery aneurysm rupture during pregnancy: A case report of maternal and fetal survival

**DOI:** 10.1016/j.ijscr.2020.09.173

**Published:** 2020-09-28

**Authors:** Manato Fujii, Suguru Yamashita, Ayako Fudono, Shuichi Yanai, Jo Tashiro, Yoshiharu Takenaka, Kazuki Yamasaki, Eisaku Ito, Yukiyoshi Masaki

**Affiliations:** a Department of Surgery, Ome Municipal General Hospital, 4-16-5 Higashi Ome, Ome-shi, Tokyo, 198-0042, Japan; b Comprehensive Reproductive Medicine, Graduate School of Medical and Dental Sciences, Tokyo Medical and Dental University, 1-5-45 Yushima, Bunkyo-ku, Tokyo, 113-8519, Japan; c Department of Radiology, Ome Municipal General Hospital, 4-16-5 Higashi Ome, Ome-shi, Tokyo, 198-0042, Japan; d Department of Surgery, St. Luke’s International Hospital, 9-1 Akashicho, Chuo-ku, Tokyo, 104-8560, Japan; e Department of Pathology, Ome Municipal General Hospital, 4-16-5 Higashi Ome, Ome-shi, Tokyo, 198-0042, Japan

**Keywords:** SAA, splenic artery aneurysm, Splenic artery aneurysm, Rupture, Pregnancy

## Abstract

**Introduction:**

Pregnancy has been demonstrated as a significant risk factor of splenic artery aneurysm (SAA) formation and rupture. However, prompt diagnosis of SAA rupture in a pregnant patient showing acute abdomen has been practically challenging in light of its rarity and vague initial presentation.

**Presentation of case:**

A 40-year-old woman (gravida 1, para 0) at 35 weeks’ gestation presented to the emergency department with upper abdominal pain and nausea. Because of fetal dysfunction, emergency caesarian section was performed by a Pfannenstiel incision. Following delivery, 400 g of hemorrhage was removed from the upper abdominal cavity. Computed tomography showed a 37-mm SAA associated with copious adjacent fluid. Although selective angiography did not demonstrate active extravasation, interventional isolation of the SAA was not performed because of multiple surrounding arteries. Relaparotomy with an upper midline incision was then performed. Sudden cardiac arrest occurred upon opening the lesser sac to irrigate clots, and cardiac massage and proximal and distal clamping of the SAA were required. Eventually, splenectomy with excision of the SAA and pancreatic tail was successfully performed, but gauze packing of the open surgical wound was required because of severe coagulopathy. Following removal of the packs and closure of the abdomen 2 days after splenectomy, the patient and infant satisfactorily recovered without sequelae.

**Discussion:**

Given continual awareness of abdominal vascular collapse during pregnancy, undelayed diagnosis and safer intervention might be achieved.

**Conclusion:**

Awareness at initial presentation and multidisciplinary efforts might be essential to achieve maternal and fetal survival in SAA rupture during pregnancy.

## Highlights

• Delayed diagnosis of SAA rupture during pregnancy occurred.
• Maternal and fetal survival following SAA rupture during pregnancy was achieved.
• Multidisciplinary efforts were relevant on treatments for SAA rupture in pregnancy.

## 1 Introduction

Splenic artery aneurysm (SAA) is the third most common type of abdominal aneurysm after aortic and iliac artery aneurysms, and it represents about 60% of visceral artery aneurysms [1]. The prevalence of SAA is reportedly <1% among the general population [2,3]; however, it is more common in women, with a female:male ratio of 4:1 [1]. Generally, intervention for SAA should be considered if it is symptomatic or at high risk of rupture [4]. Among the proposed predictors of SAA rupture, pregnancy has been demonstrated to be the most relevant factor [5]. Indeed, among the >400 cases of SAA rupture that have been described in the literature, approximately 30% occurred during pregnancy [6]. Although still controversial, changes in hormonal factors and local hemodynamics have been regarded as the main causes of SAA formation and rupture during pregnancy [4].

However, lethal complications of SAA rarely develop during pregnancy. A nationwide study in the United States showed that the estimated maternal mortality rate was 23.8 per 100,000 live births [7]. Although most cases have been attributable to obstetric causes, abdominal vascular complications have accounted for a minority of cases [8]. In fact, one study at a medical center revealed no occurrence of SAA rupture among 67,616 deliveries during a 5-year period [5], and another study at a military center showed one SAA rupture during pregnancy among 27,587 deliveries during a 6-year period (0.004% prevalence) [9]. Moreover, the principal complaints in patients with SAA rupture are nonspecific, resulting in a clinical picture similar to that of other major obstetric complications [8]. In addition, abdominal computed tomography might be initially avoided even in cases of acute abdomen because of the radiation hazards during pregnancy [10]. Consequently, 70% of cases of SAA rupture during pregnancy have been primarily misdiagnosed as uterine rupture or placental abruption [11]. Because of this diagnostic delay, the maternal and fetal mortality rates associated with SAA rupture are as high as 75% and 95%, respectively, in contrast to the general mortality rate of 25% [2,5].

Although SAA rupture is rare, its prompt consideration as a differential diagnosis when encountering acute situations in pregnancy may increase the chance of a successful outcome. We herein present a case of SAA rupture in a pregnant patient with achievement of maternal and fetal survival through the efforts of a multidisciplinary team. This case is reported to promote awareness regarding SAA rupture as a noteworthy complication during pregnancy. This case has been reported in line with the SCARE criteria [12].

## 2 Presentation of case

A 40-year-old woman (gravida 1, para 0) at 35 weeks’ gestation with a medical history of mild hypertension and no family history was admitted to the hospital with the principal complaint of upper abdominal pain and nausea. Previous pregnancy course had been uneventful, and recent conducted treatment and/or prescription, which could lead to reduced fetal heart rate, were absent. Since pallor of the face and coldness of limbs were observed despite of lack of apparent laboratory anemia (Hemoglobin 12.0 g/dL) and hypotension (blood pressure 99/74 mmHg), bleeding problem in her abdomen was suspected. Bedside ultrasound demonstrated a low fetal heart rate (<60 beats/min). Therefore, emergency cesarean section with a Pfannenstiel incision was performed 20 min after arrival based on a provisional diagnosis of uterine rupture or placental abruption despite the absence of vaginal bleeding. Following rapid delivery of a female infant weighing 2,002 g and with an Apgar score of 3 at 1 min and 5 at 5 min, 400 g of hemorrhage was found in the upper abdominal cavity; however, a gynecological origin of the hemorrhage was not found. After closure of the uterus and performance of intraperitoneal irrigation, the abdomen was closed when the bleeding had ceased. No fresh bleeding was seen through the lower abdominal incision.

**Fig. 1:**
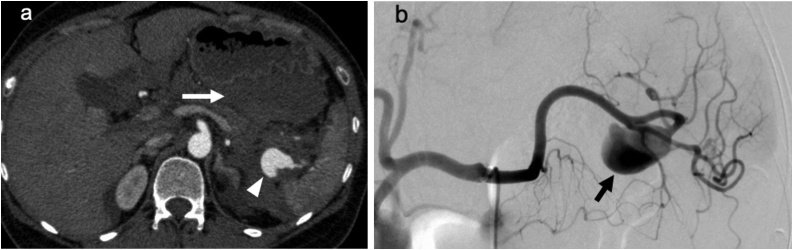
**Computed tomography and angiography images.** (a) Enhanced abdominal computed tomography showed a splenic artery aneurysm of 37 mm in diameter, which was located near the splenic hilum (arrowhead). Fluid collection was present around the splenic artery aneurysm, particularly in the lesser sac (arrow). (b) Selective angiography of the splenic artery showed the splenic artery aneurysm without apparent active extravasation (arrow). Multiple peripheral arteries were situated distal to the splenic artery aneurysm and might have been connected to the aneurysm.

For further evaluation of the intra-abdominal hemorrhage, enhanced abdominal computed tomography was promptly performed. This revealed an SAA of 37 mm in diameter located near the splenic hilum, associated with massive adjacent fluid collection (Fig. 1a). While subsequent selective angiography of the splenic artery showed an SAA with no evidence of ongoing extravasation, interventional isolation was considered an ineffective approach because multiple peripheral branches of the splenic artery were involved (Fig. 1b).

**Fig. 2:**
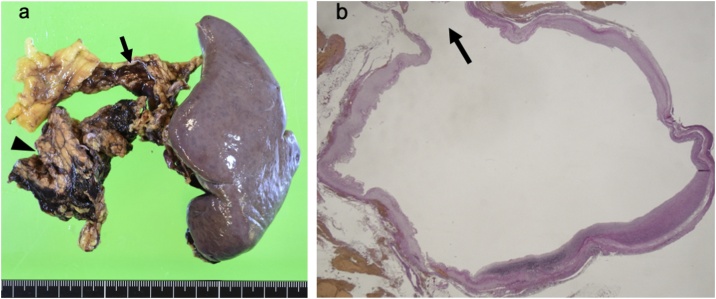
**Macroscopic and microscopic findings.** (a) Splenectomy was performed with excision of the splenic artery aneurysm (arrow) and pancreatic tail (arrowhead). (b) The wall of the splenic artery aneurysm showed complete rupture of all three vascular layers (arrow). Hematoxylin and eosin stain; original magnification, ×10.

About 6 h after arrival, the patient underwent relaparotomy with an upper midline incision. During irrigation of the space in the lesser sac, which was full of blood, sudden cardiac arrest occurred. After approximately 30 min of cardiac massage and proximal and distal clamping of the SAA, sinus tachycardia was achieved. Splenectomy with excision of the aneurysm was then performed. Because pancreatic injury occurred at the time of clamping, pancreatectomy at the small part of the tail was performed with staple closure (Fig. 2a). Additionally, the open surgical wound was packed with gauze because of generalized bleeding from the exfoliated area (estimated blood loss of approximately 2,000 g). After aggressive correction of anemia and coagulopathy and confirmation of normal neurological findings in the intensive care unit, a third operation was performed about 48 h following the gauze packing. This operation involved removal of the gauze packs, confirmation of no fresh bleeding, and closure of the abdomen. Throughout this intensive therapeutic course, the patient underwent transfusion of 30 units of red blood cells, 30 units of fresh frozen plasma, and 30 units of a platelets. Pathological examination revealed disruption of all vascular layers, confirming SAA rupture (Fig. 2b).

The patient and her daughter were eventually discharged in good condition with no neurological sequelae on postoperative day 38 and 41, respectively. Maternal recovery had been relatively complicated by a Clavien–Dindo grade IIIa pancreatic fistula. Their ambulant follows have been uneventful.

This study was carried out in accordance with the principles of the Declaration of Helsinki, and informed consent for publication was obtained from the patient.

## 3 Discussion

We have herein described a case of SAA rupture during pregnancy in which maternal and fetal survival without chronic sequelae was eventually achieved by multidisciplinary interventions. Because of lack of awareness of the SAA rupture at the initial visit, definitive treatment (splenectomy and excision of the SAA) was performed approximately 6 h after arrival, which is less than ideal. If we had accurately born in mind abdominal vascular collapse during pregnancy, an earlier diagnosis of SAA rupture might have been achieved based on a more meticulous exploration at the time of caesarian section. Some obstetricians have suggested that a midline incision rather than a Pfannenstiel incision should be primarily selected for caesarian section when intraperitoneal hemorrhage is suspected [13].

In the current case, although the patient maintained relatively stable vital signs for several hours (from caesarian section to the second laparotomy), sudden collapse occurred during irrigation of the lesser sac. This phenomenon has been described as “two-stage rupture,” meaning that the initial rupture is contained within the lesser sac for a variable number of hours before it spreads into the greater sac, leading to massive intraperitoneal hemorrhage [4]. Because of the opportune timing of this phenomenon, which reportedly occurs in approximately 25% of patients with SAA rupture during pregnancy [4], we were able to reach an accurate diagnosis and implement an effective therapeutic strategy. Given our adequate knowledge regarding this pathology of SAA rupture, we could have considered preparing a balloon occlusion catheter for insertion in the splenic artery or aorta following angiography [14,15]; this might have allowed for much safer splenectomy with excision of the SAA.

Although the incidence of SAA rupture during pregnancy is reportedly low, as mentioned above [9], cases of maternal and fetal survival following SAA rupture are even rarer. All such cases in the literature are described in case reports. We reviewed all available English-language articles of maternal and fetal survival following SAA rupture during pregnancy in the PubMed database (Table 1). These reports showed a median maternal and gestational age of 32 years and 35 weeks, respectively. All patients developed rupture during the third trimester except one patient, who developed rupture during the second trimester. Notably, 2 of 10 patients with available data developed SAA rupture as primigravidas, while multiparity has been strongly associated with SAA formation and rupture [8]. All patients presented with upper abdominal pain as one of the principal complaints, and most patients also experienced nausea and/or vomiting. In terms of medical history, three patients (including the present case) of six patients with available data had systemic hypertension, and another patient had undergone adhesiolysis with laparotomy following left nephrectomy. In the patients with SAA rupture during the third trimester, a definitive diagnosis was not reached before the intra-abdominal exploration during caesarian section. Because all patients required splenectomy by emergency laparotomy except one patient who underwent ligation of the splenic artery only, a minimally invasive approach such as endovascular intervention is unlikely to be established as a definitive treatment for SAA rupture during pregnancy. In one case, the fetus developed severe neurologic impairment [11]; however, the maternal and fetal clinical courses were successful in all remaining cases.

**Table 1:** Reports of maternal and fetal survival following splenic artery aneurysm rupture during pregnancy.

No.	Author	Year	Age (years)	Gestation (weeks)	Gravida, Para	Way of diagnosis for SAA rupture	Definitive treatment for SAA rupture
1	Gibbens [17]	1974	41	No data	0, 0	Exploration during cesarean section	Splenectomy and ligation of splenic artery
2	Algwiser [13]	1991	32	38	8, 5	Exploration by relaparotomy following cesarean section	Splenectomy
3	Al Asfar [18]	2005	33	42	0, 0	Exploration during cesarean section	Splenectomy with excision of SAA
4	Perino [19]	2012	39	35	multiparous	Exploration during cesarean section	Ligation of splenic artery
5	Corey [11]	2014	29	35	multiparous	Exploration during cesarean section	Splenectomy with excision of SAA and pancreatic tail
6	Garey [20]	2014	26	36	2, 3	Exploration by relaparotomy following cesarean section	Splenectomy
7	Heitkamp [21]	2015	26	31	2, 1	Exploration during cesarean section	Splenectomy and ligation of splenic artery
8	Jacobson [22]	2016	30	22	6, 2	CT	Splenectomy with excision of SAA, followed by elective cesarean section at 37 weeks gestation
9	Abhari [23]	2019	34	34	No data	Exploration during cesarean section	Splenectomy with excision of pancreatic tail
10	Wiener [10]	2019	30	27	1, 0	Exploration during cesarean section	Splenectomy with excision of SAA
11	Present study	2020	40	35	1, 0	CT after cesarean section	Splenectomy with excision of SAA and pancreatic tail

SAA, splenic artery aneurysm; CT, computed tomography.

The necessity of screening for SAA in selective gravidas has been discussed because of the dismal mortality rate associated with rupture [11]. However, the cost–benefit ratio of routine screening of all gravidas for SAA is unfavorable because of the rarity of the disease [16]. Further reports and evaluations of similar cases might be required to accumulate knowledge regarding additional predisposing factors during pregnancy, such as systemic hypertension. As long as definitive predictors of abdominal vascular collapse during pregnancy are uncertain due to its low incidence, we just have to keep reporting those cases to lead to disease enlightenment on both sides of patients and clinicians. When an unruptured SAA is incidentally found during pregnancy, some experts have recommended elective intervention using a minimally invasive approach (endovascular or laparoscopic) with appropriate timing [10].

## 4 Conclusions

Because of its rarity compared with several other obstetric complications, SAA rupture during pregnancy can be easily overlooked at the initial visit, which might result in a subsequently life-threatening condition. Earlier awareness of SAA rupture during pregnancy, even with a vague presentation, may further contribute to maternal and fetal survival based on successful multidisciplinary team efforts.

## Declaration of Competing Interest

The authors report no declarations of interest.

## Funding

No grant support or other funding was received.

## Ethical approval

This study was carried out in accordance with the principles of the Declaration of Helsinki, and informed consent for publication was obtained from the patient.

## Consent

Written informed consent was obtained from the patient for publication of this case report and accompanying images. A copy of the written consent is available for review by the Editor-in-Chief of this journal on request.

## Author contribution

MF, AF, SY (Yanai), and JT performed the actual treatments. MF drafted the manuscript, and SY (Yamashita) revised the manuscript. All authors read and approved the final manuscript.

## Registration of research studies

Not applicable.

## Guarantor

Manato Fujii.

## Provenance and peer review

Not commissioned, externally peer-reviewed.
